# Supplementary material for: New Inhibitors of the Human p300/CBP Acetyltransferase Are Selectively Active against the Arabidopsis HAC Proteins
Source: Int J Mol Sci. 2022 Sep 9;23(18):10446. doi: 10.3390/ijms231810446 (PMC9499386; doi:10.3390/ijms231810446)

Supporting Information

New inhibitors of the human p300/CBP acetyltransferase are selectively active against the Arabidopsis HAC proteins

Chiara Longo[1†], Andrea Lepri[1†], Andrea Paciolla[1], Antonella Messore[2], Daniela De Vita[3], Maria Carmela Bonaccorsi di Patti[4], Matteo Amadei[4], Valentina Noemi Madia[2], Davide Ialongo[2], Roberto Di Santo[2], Roberta Costi[5] and Paola Vittorioso[1*]

[1] Department of Biology and Biotechnology "Charles Darwin", Sapienza University of Rome, 00185 Rome, Italy

[2] Department of Chemistry and Technology of Drug, Sapienza University of Rome, Piazzale Aldo Moro 5, 00185 Rome, Italy

[3]Department of Environmental Biology, Sapienza University of Rome, Piazzale Aldo Moro 5, 00185 Rome, Italy

[4]Department of Biochemical Sciences, Sapienza University of Rome, Piazzale Aldo Moro 5, 00185 Rome, Italy

[5] Department of Chemistry and Technology of Drug, Instituto Pasteur, Fondazione Cenci Bolognetti, Sapienza University of Rome, Piazzale Aldo Moro 5, 00185 Rome, Italy

† These authors contributed equally to this work

* Correspondence: paola.vittorioso@uniroma1.it

Contents:

1. **Table S1. Specific primer sets used for cloning and expression analysis ……..S3**
2. **Figure S1. ^{1}H spectrum of compound 2a……………………………………………S4**
3. **Figure S2. ^{1}H spectrum of compound 2b……………………………………………S5**
4. **Figure S3. ^{1}H spectrum of compound 2c……………………………………………S6**
5. **Figure S4. ^{1}H spectrum of compound 3……………………………………………..S7**
6. **Figure S5. IR spectrum of compound 2a……………………………………………S8**
7. **Figure S6. IR spectrum of compound 2b…………………………………………...S9**
8. **Figure S7. IR spectrum of compound 2c……………………………………………S10**
9. **Figure S8. IR spectrum of compound 3……………………………………………..S11**

Table S1. Specific primer sets used for cloning and expression analysis

**Gene (ID gene)**	***Forward***	***Reverse***
Expression analysis		
UBQ10 (AT4G05320)	GGCCTTGTATAATCCTGATGAATAAG	AAAGAGATAACAGACGGAAACATAGT
GAPA1 (AT3G26650)	GCTGAGGAAGTCAACGCTGC	CGGACACTAGTGGCTCATCG
HLS1 (AT4G37580)	ACTACCTTCGATACCGTCCGT	GGATTTCACCATCTTCACCGC
ORG1 (AT5G53450)	GGGTGGGTGATGATACTACTG	CAGACCATGGCTGTGCAAGTA
LTP5 (AT3G51600)	GTGTGCATGTTAGTGACCGCT	CGCCTCGGGTCAAGTAGTTAT
LSH6 (AT1G07090)	TACCTCAAGAACCACAAGCCG	TTGGTCTTACCGAACTGGTCG
ERF4 (AT3G15210)	AAGCGTCCTTGGGGCCGTTA	CGCCTCTTCAGCCGTATCGA
ERF6 (AT4G17490)	TCGAATCCTCCTCGCGTTAC	TCGGTGGTGCGATCTTCAAC
HAC1 (AT1G79000)	GCTCCTCTTTCTACGCCATG	CGAGGATATGAACATTGAGGTG
HAC5 (AT3G12980)	ATGCTGGTGTAGGGATGAGC	CACCTGCAACTGCTCGACA
Cloning		
cl HAC1 NcoI	AAAACCATGGTTCTTGGAGCTAAAGACTTAC	
cl HAC1 XhoI		AAAACTCGAGAGCGGTTGGGTTATGTAGATG

Figure S1. ^{1}H spectrum of compound 2a

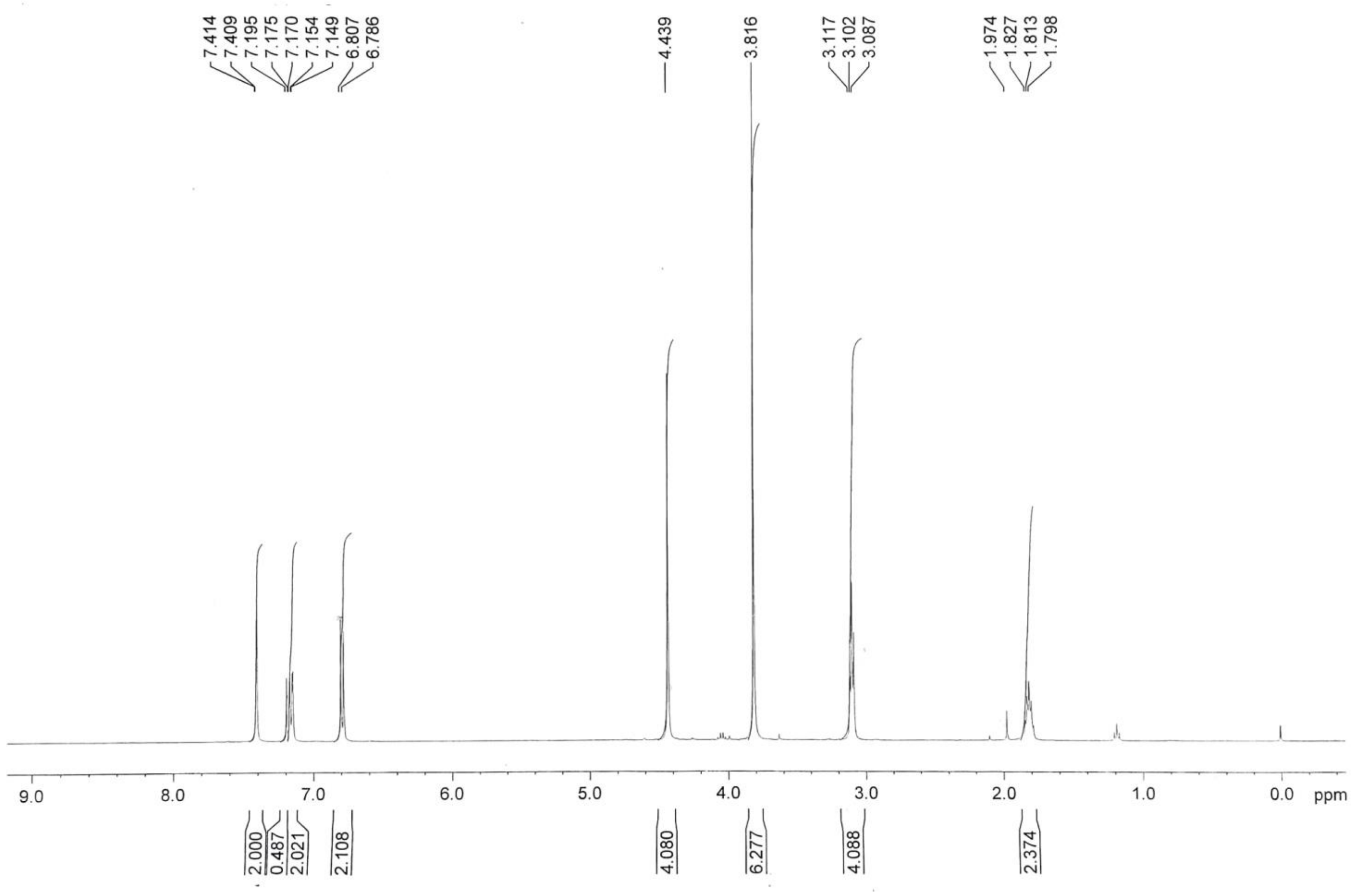

Figure S2. ¹H spectrum of compound 2b

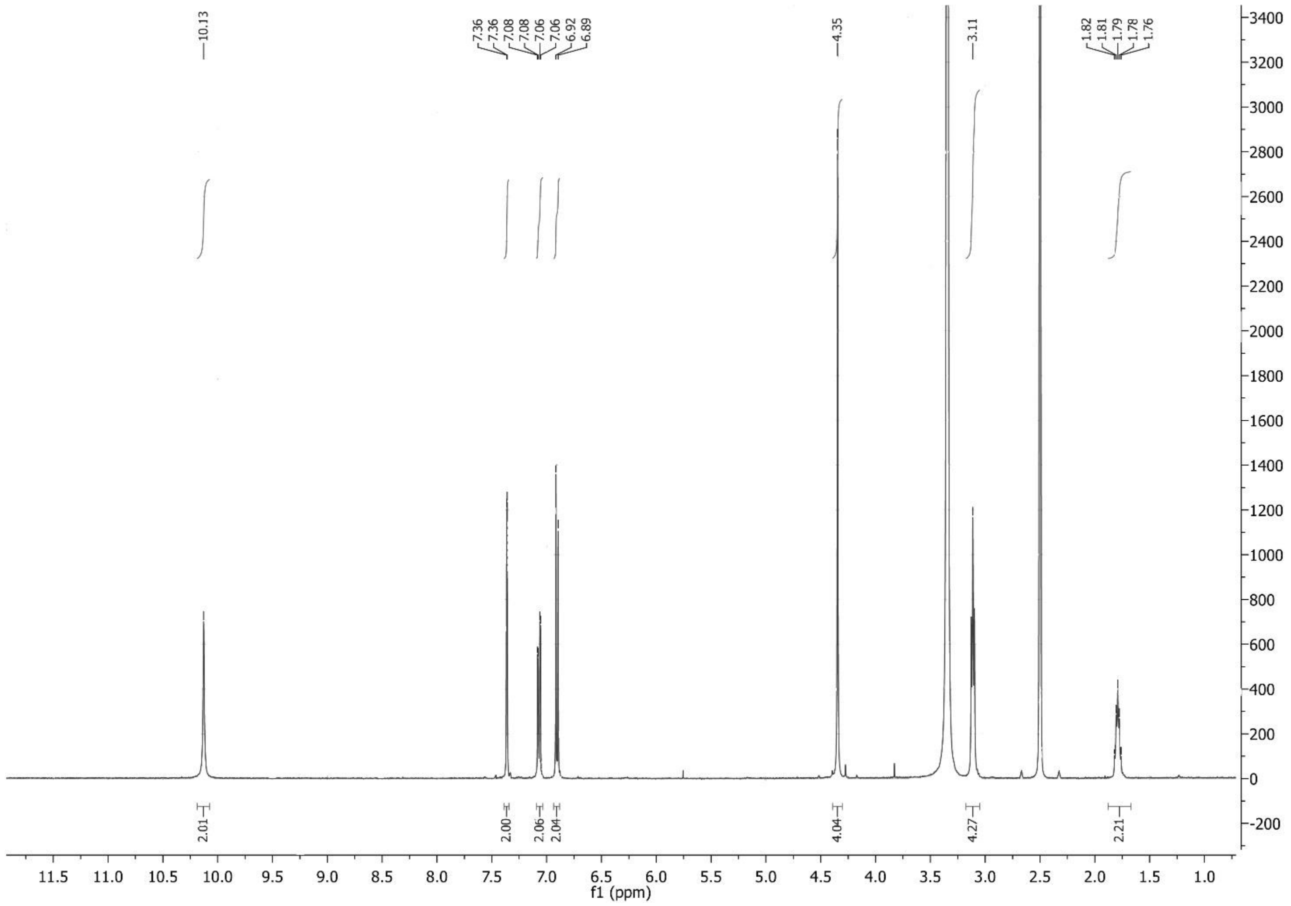

Figure S3. ^{1}H spectrum of compound 2c

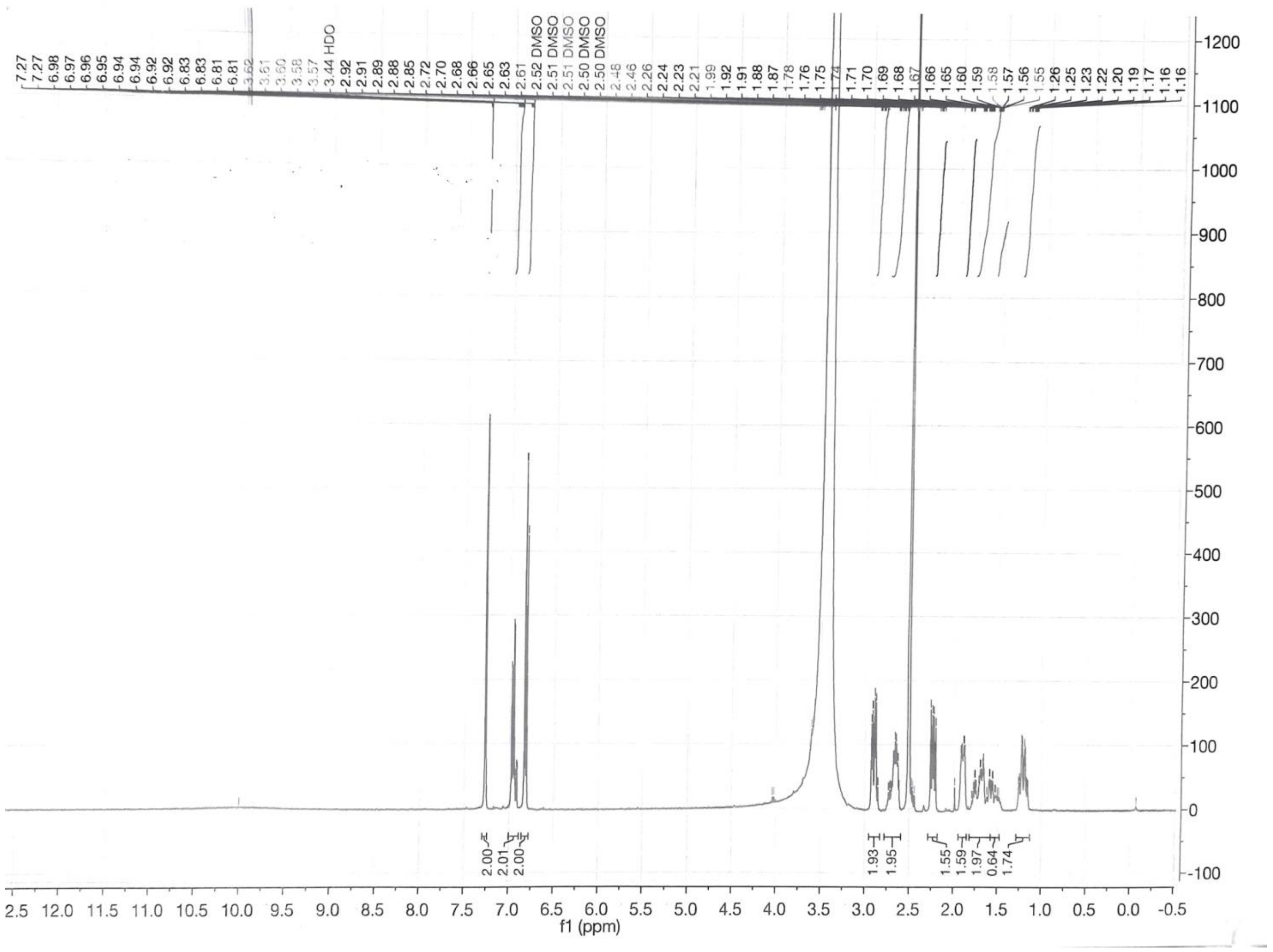

Figure S4. ¹H spectrum of compound 3

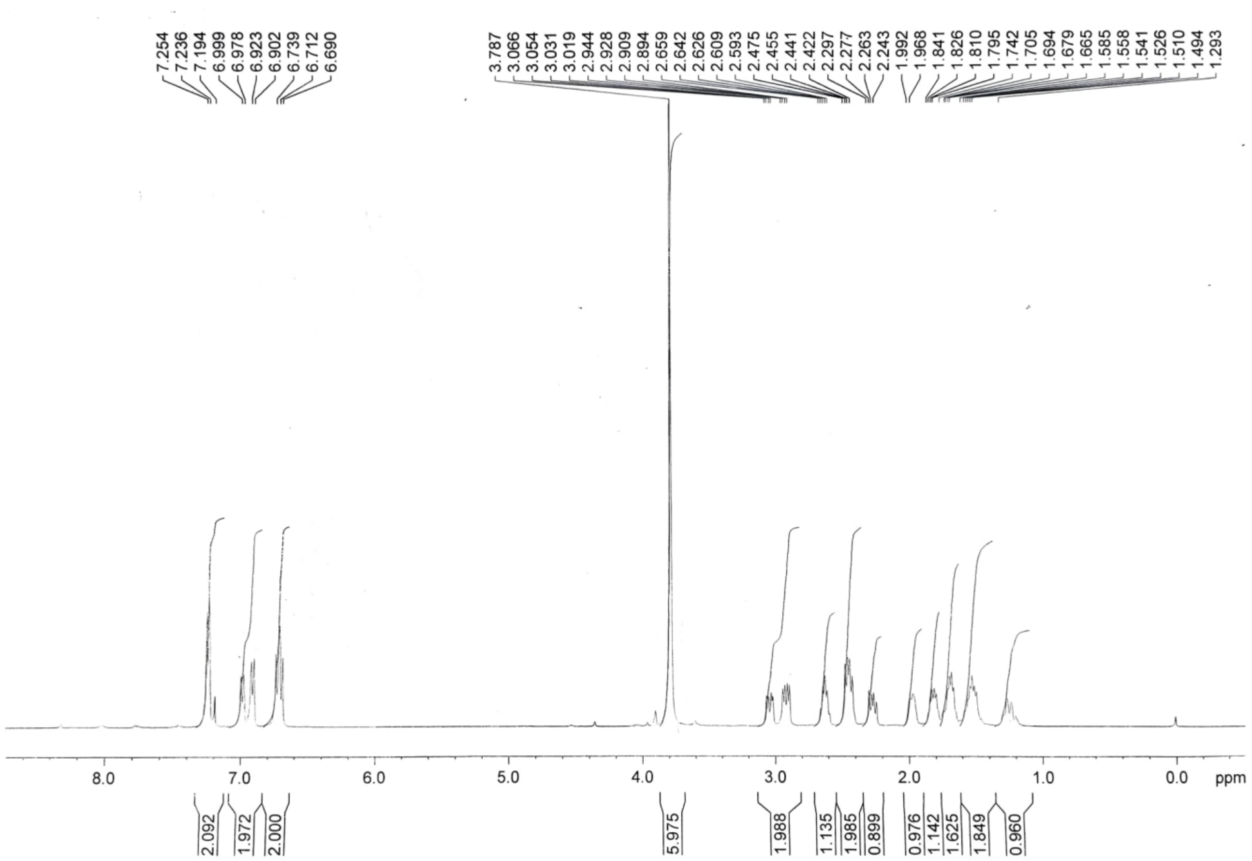

Figure S5. IR spectrum of compound 2a

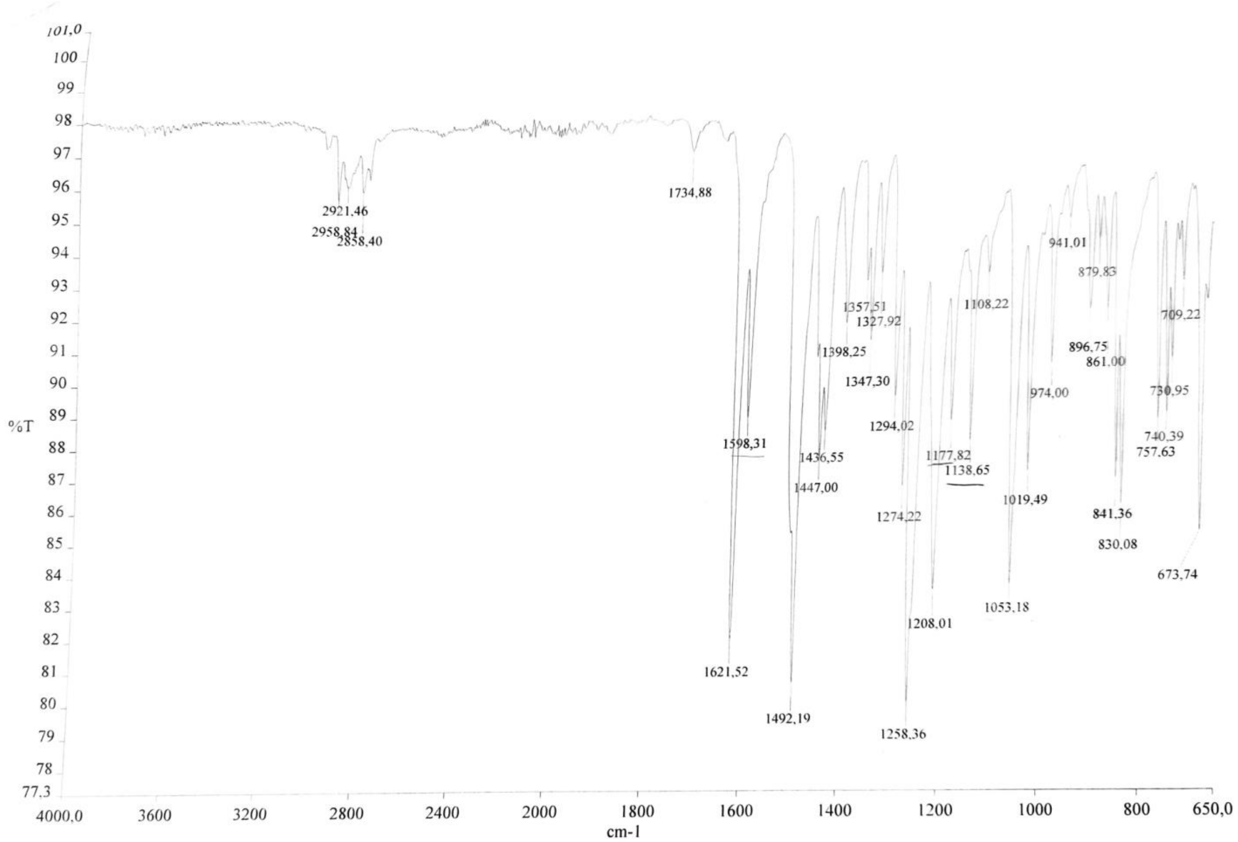

Figure S6. IR spectrum of compound 2b

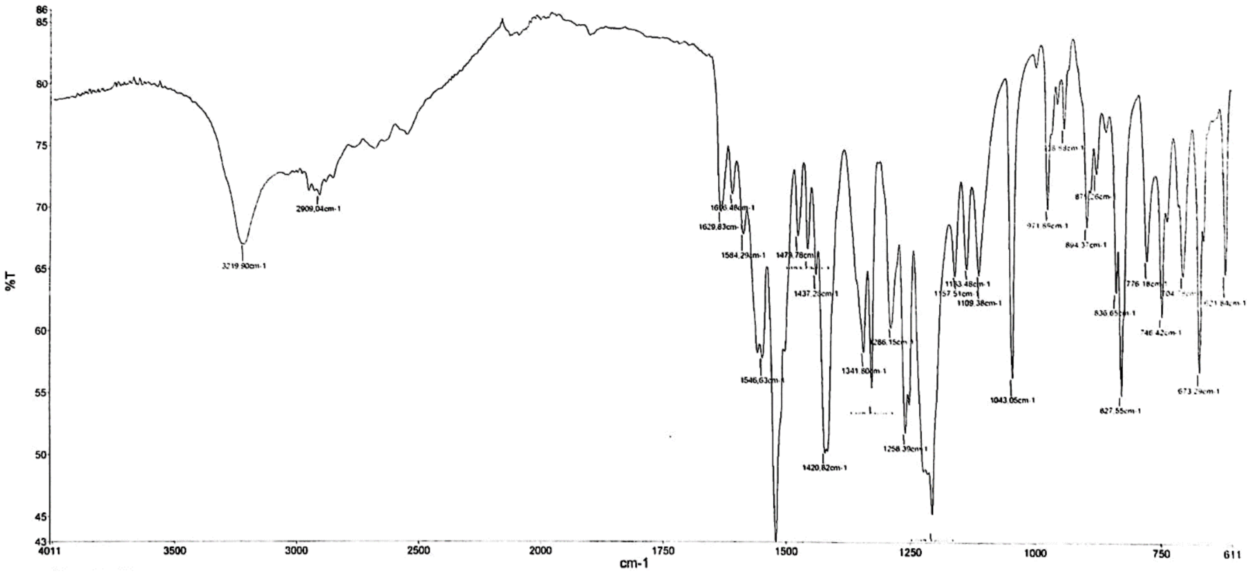

Figure S7. IR spectrum of compound 2c

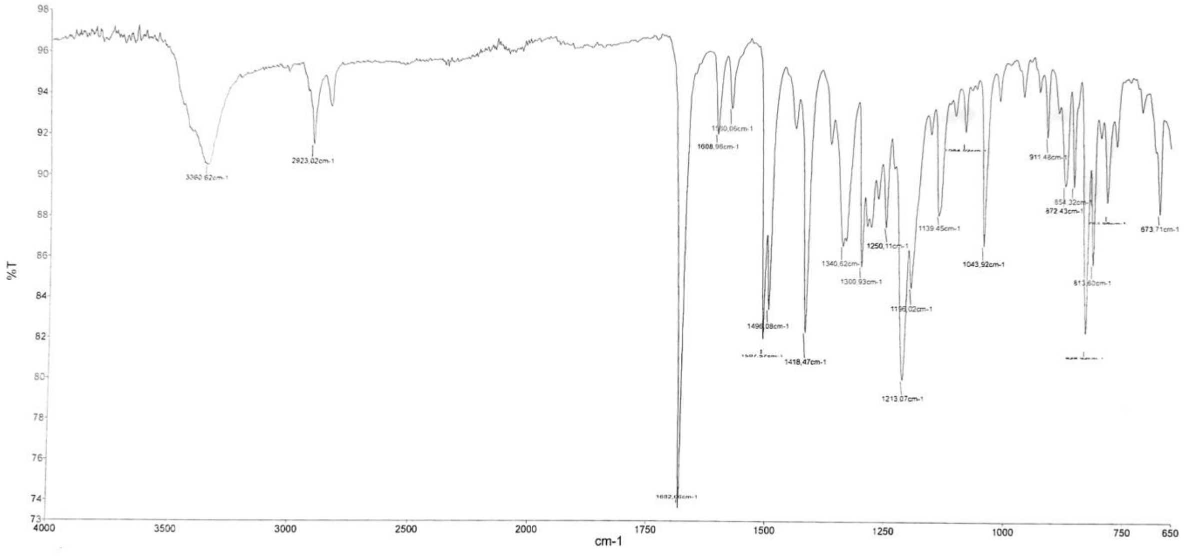

Figure S8. IR spectrum of compound 3

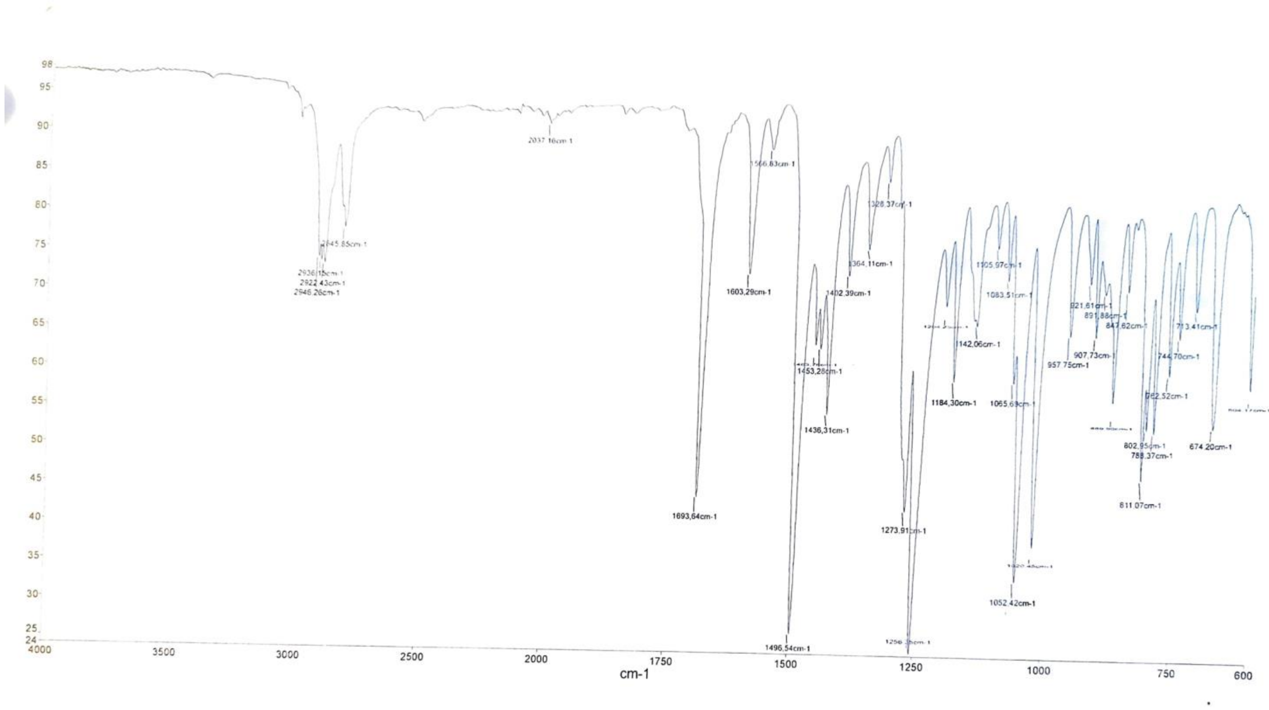

Supplement: Supplementary file 1 [file ijms-23-10446-s001.zip › ijms-1875110-supplementary.pdf]
